# Association of the Serum Levels of the Nucleocapsid Antigen of SARS-CoV-2 With the Diagnosis, Disease Severity, and Antibody Titers in Patients With COVID-19: A Retrospective Cross-Sectional Study

**DOI:** 10.3389/fmicb.2021.791489

**Published:** 2021-12-09

**Authors:** Rin Yokoyama, Makoto Kurano, Yuki Nakano, Yoshifumi Morita, Hiroko Ohmiya, Yoshiro Kishi, Jun Okada, Chungen Qian, Fuzhen Xia, Fan He, Liang Zheng, Yi Yu, Miyuki Mizoguchi, Yoshimi Higurashi, Sohei Harada, Daisuke Jubishi, Koh Okamoto, Kyoji Moriya, Tatsuhiko Kodama, Yutaka Yatomi

**Affiliations:** ^1^Department of Clinical Laboratory, The University of Tokyo Hospital, Tokyo, Japan; ^2^Department of Clinical Laboratory Medicine, Graduate School of Medicine, The University of Tokyo, Tokyo, Japan; ^3^Sales and Marketing Division, Business Planning Department, Medical and Biological Laboratories Co., Ltd., Tokyo, Japan; ^4^The Key Laboratory for Biomedical Photonics of MOE at Wuhan National Laboratory for Optoelectronics-Hubei Bioinformatics and Molecular Imaging Key Laboratory, Systems Biology Theme, Department of Biomedical Engineering, College of Life Science and Technology, Huazhong University of Science and Technology, Wuhan, China; ^5^Reagent R and D Center, Shenzhen YHLO Biotech Co., Ltd., Guangdong, China; ^6^Department of Infection Control and Prevention, The University of Tokyo, Tokyo, Japan; ^7^Laboratory for Systems Biology and Medicine, Research Center for Advanced Science and Technology, The University of Tokyo, Tokyo, Japan

**Keywords:** COVID-19, coronavirus disease 2019, N antigen, nucleocapsid antigen, severity, diagnosis, antibody titer

## Abstract

**Background:** Several types of laboratory tests for COVID-19 have been established to date; however, the clinical significance of the serum SARS-CoV-2 nucleocapsid (N) antigen levels remains to be fully elucidated. In the present study, we attempted to elucidate the usefulness and clinical significance of the serum N antigen levels.

**Methods:** We measured the serum N antigen levels in 391 serum samples collected from symptomatic patients with a confirmed diagnosis of COVID-19 and 96 serum samples collected from patients with non-COVID-19, using a fully automated chemiluminescence immunoassay analyzer.

**Results:** Receiver operating characteristic analysis identified the optimal cutoff value of the serum N antigen level (cutoff index, based on Youden’s index) as 0.255, which yielded a sensitivity and specificity for the diagnosis of COVID-19 of 91.0 and 81.3%, respectively. The serum N antigen levels were significantly higher in the patient groups with moderate and severe COVID-19 than with mild disease. Moreover, a significant negative correlation was observed between the serum N antigen levels and the SARS-CoV-2 IgG antibody titers, especially in patients with severe COVID-19.

**Conclusion:** Serum N antigen testing might be useful both for the diagnosis of COVID-19 and for obtaining a better understanding of the clinical features of the disease.

## Introduction

Coronavirus disease 2019 (COVID-19), caused by severe acute respiratory syndrome coronavirus 2 (SARS-CoV-2), which was declared as a pandemic in early 2020, is still spreading and causing deaths and morbidity around the world in 2021 (World Health Organization, 2021). Several types of laboratory tests have been established for the diagnosis of COVID-19, including tests involving nucleic acid amplification (e.g., reverse transcription polymerase chain reaction, RT-PCR), antigen tests to detect specific components [mainly nucleocapsid antigen (N) antigen] of the SARS-CoV-2 virus, and antibody tests to detect antibodies against SARS-CoV-2. At present, RT-PCR testing is used as the gold standard for diagnosing COVID-19 (Corman et al., 2020); antigen testing has also been demonstrated to show almost similar diagnostic ability to RT-PCR testing and is used in the clinical diagnosis of SARS-CoV-2 infection (Hirotsu et al., 2020). In regard to antibody tests, they show high diagnostic sensitivity and specificity a few weeks after the onset of symptoms, while the diagnostic sensitivity is relatively low in the early phase after symptom onset (Miller et al., 2020; Nakano et al., 2021).

In addition to being useful for the diagnosis, some of these laboratory parameters have also been reported to be related to the severity of the disease. In regard to RT-PCR and antigen testing, although the number of studies is limited, it has been reported that the viral load in upper respiratory tract specimens measured using the RT-PCR test or antigen tests is associated with the severity of COVID-19 (Fajnzylber et al., 2020). In regard to antibody tests, while some studies contend that the titers of antibodies against SARS-CoV-2 do not show positive associations with the disease severity (Gozalbo-Rovira et al., 2020; Kong et al., 2020; Phipps et al., 2020), numerous other studies have demonstrated the existence of associations between SARS-CoV-2 antibody titers and the severity of COVID-19 (Röltgen et al., 2020; Bläckberg et al., 2021; De Donno et al., 2021; Fu et al., 2021; Legros et al., 2021; Lu et al., 2021; Patil et al., 2021; Shrivastava et al., 2021).

In addition to sputum, nasopharyngeal swab, nasal swab and saliva specimens, blood samples (serum and plasma) are promising for antigen testing, especially as collection of blood samples is associated with a much lower risk of contracting the infection during sampling. A few reports to date have demonstrated that the nucleocapsid antigen (N antigen) of SARS-CoV-2 can be detected in the plasma by enzyme-linked immunosorbent assay, chemiluminescence enzyme immunoassay, and a highly sensitive single molecule array immunoassay, although the clinical significance of the N antigen in blood samples of COVID-19 patients is not yet fully understood (Ogata et al., 2020; Belogiannis et al., 2021; Deng et al., 2021; Le Hingrat et al., 2021; Perna et al., 2021; Shan et al., 2021). Considering that several previous studies have demonstrated that SARS-CoV-2 RNA titers in blood samples are associated with the severity of COVID-19 (Eberhardt et al., 2020; Fajnzylber et al., 2020; Wölfel et al., 2020; Di Cristanziano et al., 2021), antigen testing in blood samples might be useful for predicting the severity of COVID-19 as well as for diagnosing the disease.

Taking into account the studies described above, in the present study, we measured the serum levels of N antigen levels using an automated chemiluminescence immunoassay (CLIA) analyzer, with the aim of investigating the usefulness and clinical significance of measurement of the serum N antigen levels in patients with COVID-19.

## Materials and Methods

### Subjects

We enrolled a total of 101 patients with a confirmed diagnosis of COVID-19 who were hospitalized at The University of Tokyo Hospital from April 2020 to January 2021. COVID-19 cases were defined as follows: patients with acute respiratory infection syndrome in whom SARS-CoV-2 RNA had been detected at least once in a throat swab or sputum specimen. In addition, we also enrolled 96 patients with non-COVID-19, on the basis of the presence of respiratory symptoms, history of overseas travel, or history of close contact with a confirmed COVID-19 case, in whom there was no evidence of clinical progression and the RT-PCR test was negative. The characteristics of the enrolled subjects are described in Supplementary Table S1. We classified the patients with a confirmed diagnosis of COVID-19 into three maximum severity groups: the group with mild disease, consisting of patients who did not require oxygen supplementation, the group with moderate disease, consisting of patients who required oxygen supplementation, but not mechanical ventilatory support, and the group with severe disease, consisting of patients who required mechanical ventilatory support.

The current study was performed in accordance with the ethical guidelines established in the Declaration of Helsinki. Written informed consent for sample analysis was obtained from some of the patients. For the remaining participants, from whom written informed consent could not be obtained because they had been discharged or transferred from the hospital, informed consent was obtained in the form of an opt-out on the website: patients were informed about the study on the website and those who were unwilling to be enrolled in the study were excluded.^1^,^2^ If the participants were minor, their parents or guardians could waive participating this study on behalf of them. The study design was approved by the University of Tokyo Medical Research Center Ethics Committee, which waived the need for written informed consent in cases where written informed consent could not be obtained, because only archived specimens were used and the data for this retrospective study were retrieved from the medical records (2019300NI-4 and 2020206NI).

### Samples

We collected residual serum samples of confirmed COVID-19 and non-COVID-19 patients after routine clinical laboratory testing and kept them frozen at −80°C until analysis, as previously described (Yokoyama et al., 2021). We obtained a total of 391 serum samples from the 101 patients with a confirmed diagnosis of COVID-19 (COVID-19 cases) between 1 and 16 days after symptom onset and five serum samples collected before the onset of symptoms (Supplementary Table S1). The serum samples before the onset of the infection were collected by chance since all serum samples investigated in our laboratory were stored for 3 weeks from the day when the routine laboratory testing was performed. One of the subjects of whom we collected the serum samples before the onset of COVID-19 symptoms had been hospitalized in the University of Tokyo Hospital and confirmed SARS-CoV-2 PCR negative. In other four subjects, those serum samples were collected to treat their chronic diseases and symptoms of cold were not described in medical records then. One serum sample each was used for the 96 non-COVID-19 patients (non-COVID-19 cases).

### Measurement of SARS-CoV-2 Ag and SARS-CoV-2 IgM, IgG, and Neutralizing Antibody

Antigen testing was performed using iFlash-2019-nCoV Antigen kits and an iFlash3000 fully automated CLIA analyzer supplied by Shenzhen YHLO Biotech Co., Ltd. (Shenzhen, China). The details of the assay method are described in a previous paper (Deng et al., 2021). In brief, a sandwich complex was formed between anti-N antigen antibody-coated paramagnetic beads and acridinium ester-labeled anti-N antigen antibody, if N antigen existed in the sample. Next, under a magnetic field, nonmagnetic materials were washed out with a wash buffer. Then, the chemiluminescence reaction observed after the addition of trigger solutions was measured in relative light units (RLU). Finally, the results were determined as a cutoff index (COI) by a calibration curve generated by 2-point calibration. In accordance with the manufacturer’s instructions, the cutoff value for a positive N antigen result was deemed as 1 COI in a nasal swab sample. The curves of dilution showed good linearly regardless of the presence of the SARS-CoV-2 IgM and IgG (Supplementary Figure S1 and Supplementary Table S2), suggesting that obvious interference of SARS-CoV-2 antibodies might not exist in the N antigen measurement.

The procedure for antibody testing has been described previously by some researchers (Qian et al., 2020). In brief, the IgM or IgG titers against N and spike antigen of SARS-CoV-2 in 5 μl of the sample were calculated as RLU using the analyzer and described as arbitrary units per milliliter (AU/ml) by comparing the RLU detected by the iFlash optical system with the cutoff calculated from the SARS-CoV-2 IgM or IgG calibrators containing anti-SARS-CoV-2 IgM or IgG chimeric antibody. This system was validated by us in a previous study (Yokoyama et al., 2021).

Measurement of neutralizing antibody (NAb) titer against SARS-CoV-2 was performed using iFlash-2019-nCoV NAb kit. If NAbs present in 40 μl of the sample, they reacted with receptor binding domain (RBD) of spike protein of SARS-CoV-2 antigen coated on paramagnetic microparticles to form a complex. Next, the acridinium-ester-labeled angiotensin converting enzyme 2 (ACE2) conjugate was added to competitively bind to the RBD-coated particles, which have not been neutralized by the NAbs. Then, under a magnetic field, magnetic particles were adsorbed to the wall of the reaction tube and unbound materials were washed out with a wash buffer. Finally, the chemiluminescence reaction observed after the addition of trigger solutions was measured in RLU and the results were determined as AU/ml by a calibration curve generated by 4-point calibration. In accordance with the manufacturer’s instruction, the cutoff value for positive neutralizing antibody result was deemed as 10 AU/ml.

### Real-Time RT-PCR for SARS-CoV-2 RNA

The procedure for real-time RT-PCR to quantitate cycle threshold (Ct) values for SARS-CoV-2 RNA has been described previously by us (Mizoguchi et al., 2021).

### Statistical Analysis

All statistical data were analyzed using IBM SPSS Statistics 27 (New York, United States). Mann–Whitney’s U test was used for comparisons between two groups. Dunn–Bonferroni procedure was used for comparisons among more than three groups. Spearman’s rank correlation coefficient was calculated to evaluate the correlations between the serum N antigen levels and Ct values in nasopharyngeal swab or serum antibody titers. A value of *p* < 0.05 was regarded as being indicative of statistical significance in all the analyses.

## Results

### Successful Detection of SARS-CoV-2 Ag in the Serum

**Figure 1:**
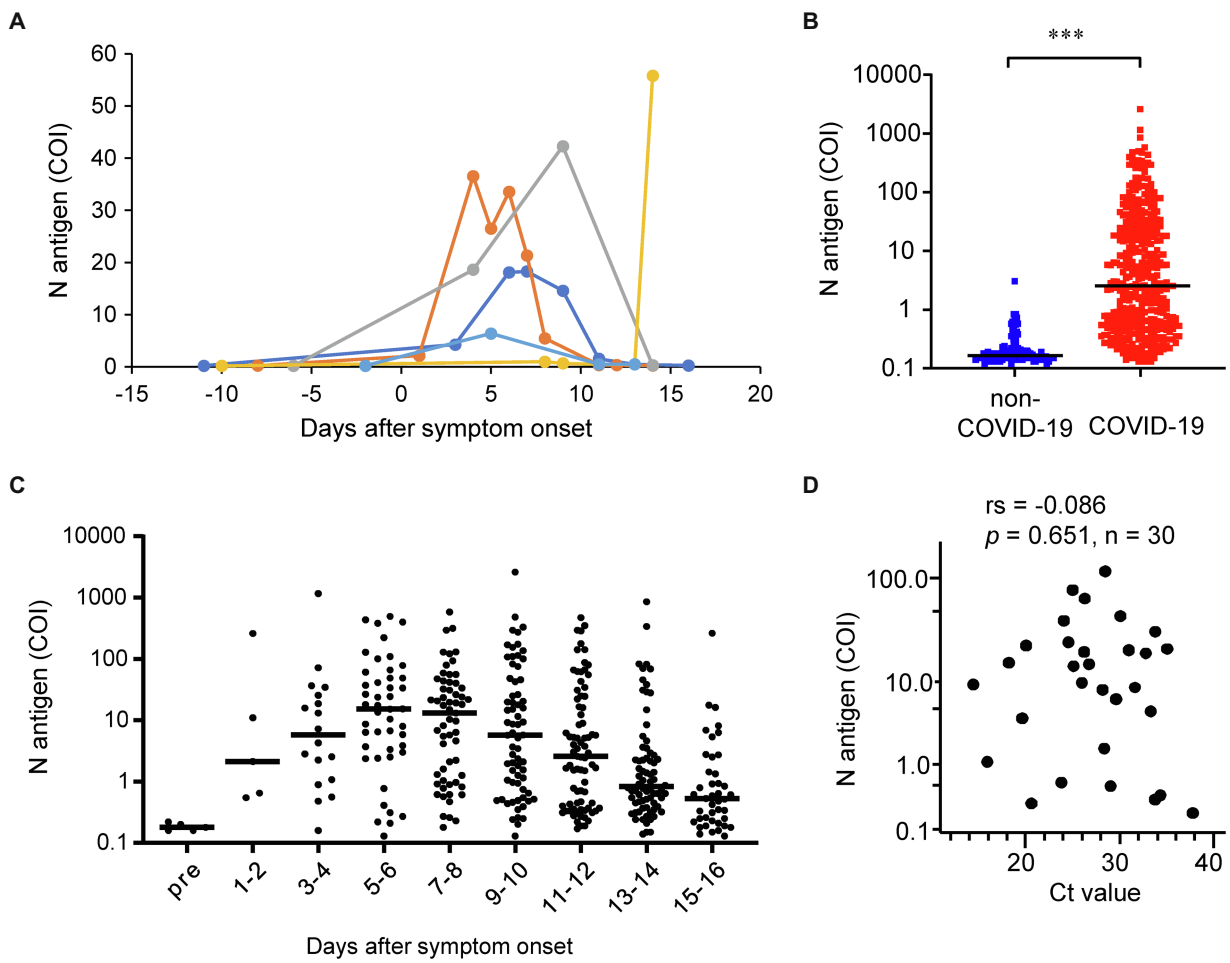
Distributions of the serum N antigen levels. **(A)** The time-courses of the serum N antigen levels are shown for the five cases for whom both serum samples before and after the onset of COVID-19 symptoms were available. **(B)** We measured the serum N antigen levels in 391 serum samples collected from 101 COVID-19 cases and one serum sample each collected from 96 non-COVID-19 cases. ***p < 0.005 **(C)** The dot plots show the time-courses of the serum N antigen levels from before (*n* = 5) through various time-points after (*n* = 391) symptom onset. The bars show the median levels at each time point. **(D)** We compared the serum N antigen levels with the Ct values of SARS-CoV-2 RNA in the same subjects on the same day as the collection of serum samples (*n* = 30). rs, spearman’s rank correlation coefficient.

First, to confirm that our measurement system could successfully detect SARS-CoV-2 antigen in serum samples, we measured the serum N antigen levels in the sera collected before and after the onset of COVID-19 symptoms in five COVID-19 cases. As shown in Figure 1A; Supplementary Figure S2, serum N antigen was below the detection limit prior to the symptom onset in all five cases. In cases 1–4, the serum N antigen level increased gradually until several days after the onset of symptoms and returned to the baseline by 10 days after symptom onset, while in case 5, the serum N antigen level remained at the baseline until it increased markedly on day 14 after symptom onset.

Second, we measured the serum N antigen levels in the 391 serum samples collected from 101 COVID-19 cases and compared them with the levels measured in one sample each collected from the 96 non-COVID-19 cases (Figure 1B). The N antigen levels in the serum samples obtained from the COVID-19 cases were significantly higher than those in the samples of the non-COVID-19 cases. Moreover, the serum N antigen levels in all but one of the non-COVID-19 cases were lower than 1 COI, which was claimed as the cutoff value for nasal swab specimens by the manufacturer. In regards to the time-course of the serum N antigen levels, the levels reached the peak at 5–6 days after the onset of symptoms, decreasing gradually thereafter (Figure 1C).

Third, we investigated the correlation between the serum N antigen level and the viral load in nasopharynx and we observed that the Spearman’s rank correlation coefficient was not significant (Figure 1D), suggesting that serum N antigen levels might have unique clinical characteristics, compared with the laboratory tests using the upper respiratory specimens.

### ROC Analysis and Optimal Cutoff Value of Serum N Antigen Levels

Next, we performed a receiver operating characteristic (ROC) analysis using data from all the 487 samples measured (COVID-19 cases, *n* = 391 and non-COVID-19 cases, *n* = 96), after excluding the samples collected prior to the onset of COVID-19 symptoms (Table 1). The area under the curve (AUC) for serum N antigen was 0.932 (95% Cl, 0.908–0.957). The optimal cutoff value was identified as 0.255 COI, based on Youden’s index. Furthermore, we conducted ROC analysis using samples classified by the sampling day after symptom onset (Table 1). From 1 to 6 days after the onset of symptoms onset, the diagnostic specificity for COVID-19 was 99.0% when the cutoff value was set at 0.865 COI based on Youden’s index.

**Table 1:** The time courses of the diagnostic values determined by ROC analysis.

Days after symptom onset	Days 1–6	Days 1–12	Days 1–16
Number of subjected used for the analyses as COVID-19 cases			
Samples (number)	69	275	391
Cases (number)	43	91	101
ROC analysis			
AUC (95% Cl)	0.957 (0.923–0.991)	0.954 (0.934–0.975)	0.932 (0.908–0.957)
*p* value	<0.005	<0.005	<0.005
Cutoff value (COI)	0.865	0.255	0.255
Sensitivity (%)	82.6	94.5	91.0
Specificity (%)	99.0	81.3	81.3

*We performed the ROC analysis. The cutoff values were determined based on Youden’s index. We used 96 non-COVID-19 subjects as a negative control group*.

**Figure 2:**
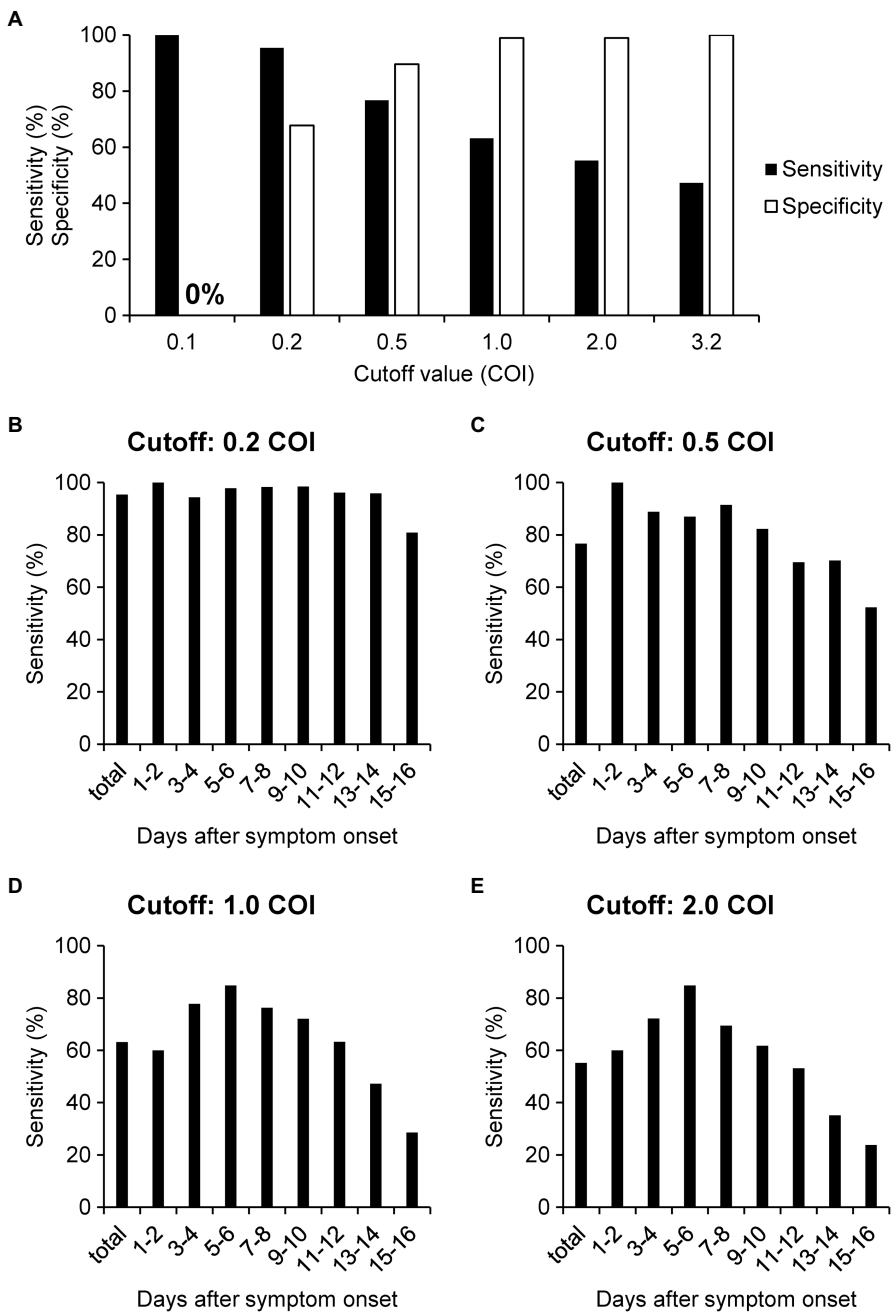
Sensitivity and specificity of N antigen testing for the diagnosis of COVID-19. **(A)** We calculated the sensitivity and specificity using the serum samples of patients with non-COVID-19 cases (*n* = 96) and COVID-19 (*n* = 391), with the cutoff value set at 0.2, 0.5, 1.0, 2.0, or 3.2 COI. **(B–E)** We calculated the sensitivity for each time-point after symptom onset using serum samples collected from COVID-19 cases when the cutoff value was set at 0.2, 0.5, 1.0, and 2.0 COI. The numbers of samples at each time point are indicated in Supplementary Table S1.

We investigated the modulation of the diagnostic sensitivity and specificity for various cutoff values (Figure 2A). As compared to that obtained for a cutoff value of 0.2 COI, the diagnostic specificity increased to nearly 99% when the cutoff value was set at 1.0 COI. At 3.2 COI, the specificity reached 100%. As shown in Figure 2A, the sensitivity decreased when the specificity improved. Next, we examined the modulation of the diagnostic sensitivity at each time-point after symptom onset when the cutoff value was set at 0.2, 0.5, 1.0, and 2.0 COI (Figures 2B–E). When the cutoff was set at 1.0 COI, the highest sensitivity (84.8%) was obtained on days 5–6 after the symptom onset.

To further validate the optimal cutoff value of serum N antigen, we calculated the overall concordance rate between the results of serum N antigen and those of RT-PCR for SARS-CoV-2 RNA in nasopharyngeal swab, a present standard diagnostic method for COVID-19 in Japan, when the cutoff values were modified (Supplementary Table S3). The overall concordance rate was the highest at days 1–4 after the symptom onset when the cutoff value was set at 0.5 COI. At days 5–10 after the symptom onset, the optimal cutoff value was 0.255 or 1.0 COI. After 11 days, the overall concordance rates decreased.

### Association of the Serum N Antigen Levels With the Severity of COVID-19

**Figure 3:**
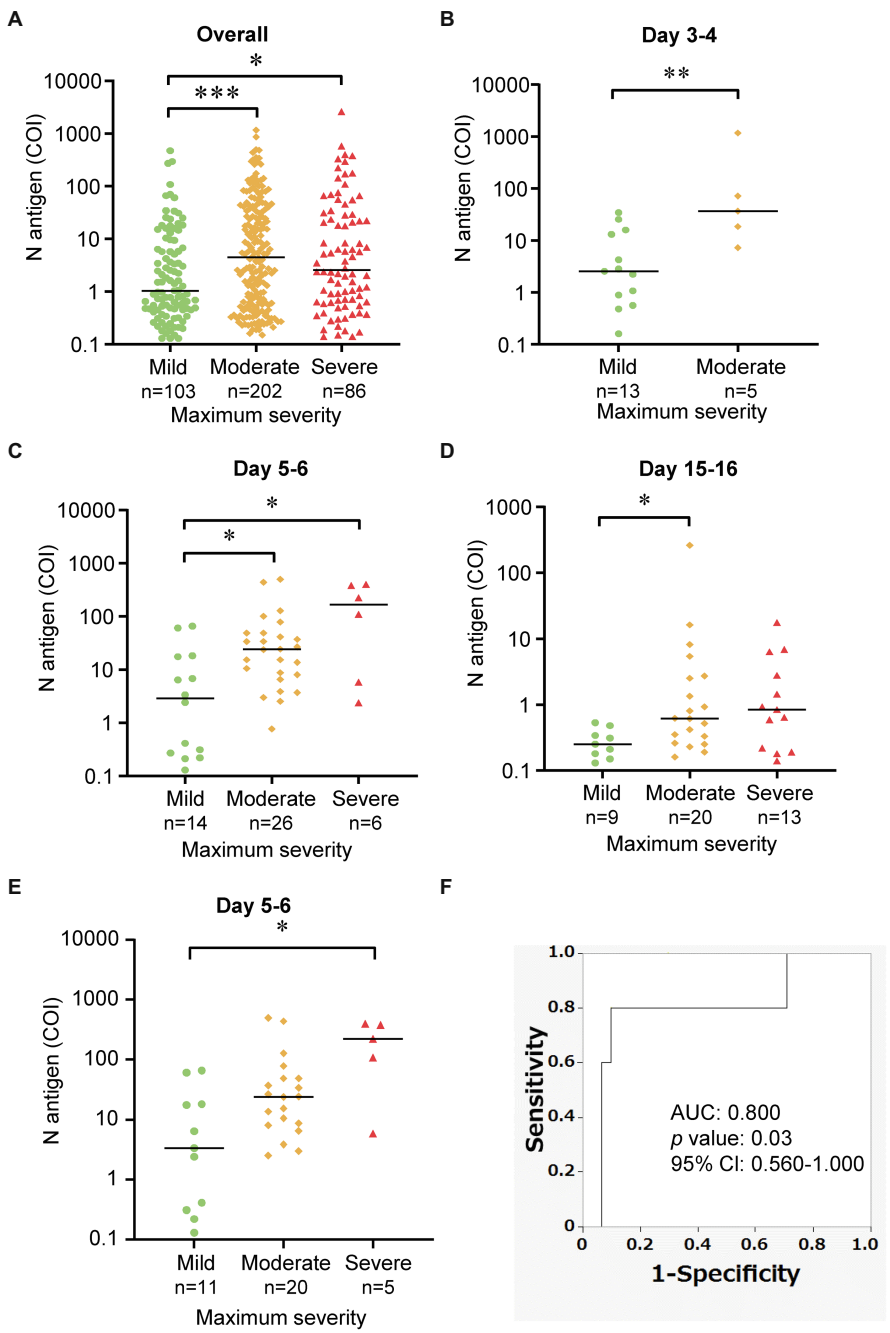
Comparison of the serum N antigen levels among the three maximum severity groups. These dot plots show a comparison of the serum N antigen levels among the mild, moderate and severe group **(A)** overall, **(B–D)** at various time-points after symptom onset, and **(E)** using one time-point serum N antigen levels from individual patients at day 5–6. The bars show the median levels at each time-point. **(F)** We performed ROC analysis to discriminate the group with severe COVID-19 from other groups. *p < 0.05; **p < 0.01; ***p < 0.005.

Next, to evaluate the association between the serum N antigen levels and the maximum disease severity, we compared the serum N antigen levels among the groups with mild, moderate, and severe COVID-19, classified as described in the “Materials and Methods” section. As shown in Figure 3A, the serum N antigen levels in the groups with moderate and severe COVID-19 were significantly higher than those in the group with mild disease. We further performed sub-analyses as shown in Supplementary Figure S3. As shown in Figures 3B–D, the serum N antigen levels in the group with moderate COVID-19 were significantly higher than those in the group with mild disease on days 3–4, days 5–6, and days 15–16 after the onset of symptoms. We observed no significant differences in the serum N antigen levels between the groups with moderate and severe disease at each time-point of measurement.

To elucidate the predicting ability of the serum N antigen levels for the disease severity, we investigated the association between the representative serum N antigen levels of individual subjects at the specific time point of day 5 or day 6 (36 samples from 36 subjects) and the maximum COVID-19 severity. We observed that the serum N antigen levels in the groups with severe COVID-19 were significantly higher than those in the group with mild severity. Furthermore, as shown in Figure 3F, we performed a ROC analysis to distinguish the subjects with severe COVID-19 from those with the maximum severity of mild and moderate. The AUC for serum N antigen was 0.800 (95% Cl, 0.560–1.000) and the sensitivity for severe COVID-19 was 80% and the specificity was 90.3% when the cutoff value was set at 93.505 COI, based on Youden’s index (Figure 3F). We showed the time course of serum N antigen of the cases of 13 patients, the severity of whom had progressed between days 1–16 after the symptom onset (Supplementary Figure S4). As shown in Supplementary Figure S4, in 12 of 13 patients, the peaks of serum N antigen level were observed before or on the same day as the maximum severity.

### Correlations Between the Serum N Antigen Levels and Serum Anti-SARS-CoV-2 Antibody Titers

**Figure 4:**
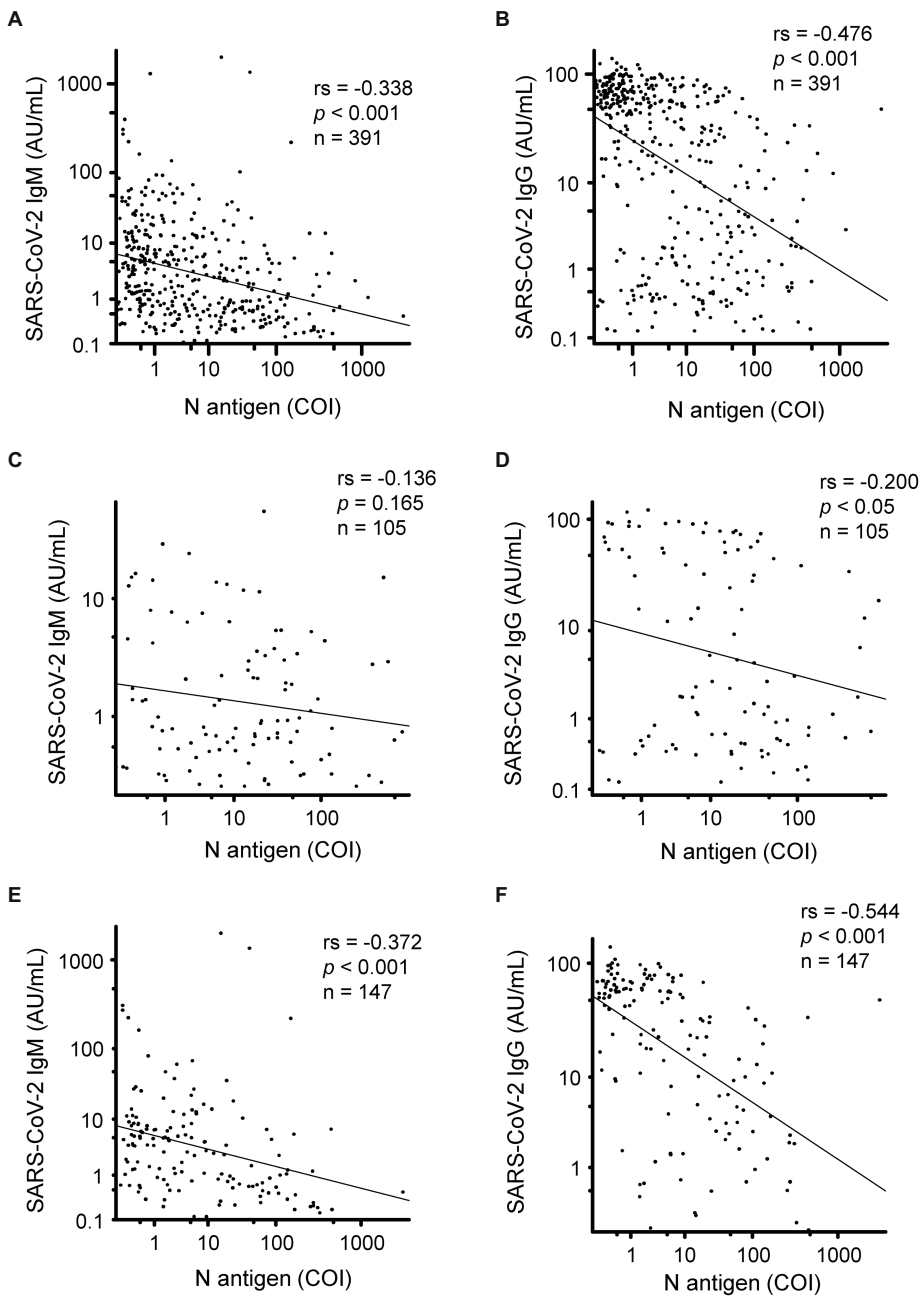
Correlation of the serum N antigen levels with the SARS-CoV-2 specific antibody titers. We compared the serum N antigen levels in relation to the SARS-CoV-2 IgM/IgG titers in samples collected between 1 and 16 days **(A,B)**, 5 and 8 days **(C,D)**, and 9 and 12 days **(E,F)** after symptom onset. rs, spearman’s rank correlation coefficient.

Finally, we investigated the correlations between the serum N antigen levels and SARS-CoV-2 specific antibody titers (Table 2). When data from all the measured samples were included in the analyses, significantly negative correlations were observed between the SARS-CoV-2 IgM or IgG titers and the serum N antigen levels in the samples collected 1–16 days after the onset of symptoms (Figures 4A,B). A significant negative correlation between the serum N antigen levels and SARS-CoV-2 IgG, but not SARS-CoV-2 IgM, titers was observed between days 5 and 8 after symptom onset (Figures 4C,D). On the other hand, between days 9 and 12, significantly negative correlations were observed between the serum N antigen levels and both SARS-CoV-2 IgG and IgM titers (Figures 4E,F). Moreover, the group with severe COVID-19 showed the strongest negative correlation between the serum N antigen levels and SARS-CoV-2 IgG titers or NAb titers among the three groups with mild, moderate and severe COVID-19 (Table 2).

**Table 2:** Correlation coefficients between the serum N antigen levels and the SARS-CoV-2 IgM/IgG titers.

Days after symptom onset	Maximum Severity	N	v.s. SARS-CoV-2 IgM		v.s. SARS-CoV-2 IgG		v.s. SARS-CoV-2 NAb		rs	*p*	rs	*p*	rs	*p*
1–4	Mild	15	−0.196	0.483	−0.238	0.394	−0.121	0.666
	Moderate	8	0.357	0.385	−0.286	0.493	−0.095	0.823
	Severe	No sample						
	Total	23	−0.049	0.825	−0.14	0.524	−0.119	0.59
5–8	Mild	31	−0.255	0.166	−0.113	0.547	−0.097	0.605
	Moderate	59^*^	−0.283	0.03	−0.492	<0.001	−0.399	0.002
	Severe	15	−0.396	0.231	−0.825	<0.001	−0.682	0.005
	Total	105^*^	−0.136	0.165	−0.2	0.04	−0.22	0.025
9–12	Mild	32	−0.169	0.356	−0.211	0.246	−0.345	0.053
	Moderate	80	−0.542	<0.001	−0.684	<0.001	−0.702	<0.001
	Severe	35	−0.393	0.02	−0.817	<0.001	−0.766	<0.001
	Total	147	−0.372	<0.001	−0.544	<0.001	−0.516	<0.001
13–16	Mild	25	−0.24	0.249	−0.301	0.143	−0.356	0.081
	Moderate	55	−0.228	0.094	−0.382	0.004	−0.463	<0.001
	Severe	36	0.24	0.92	−0.664	<0.001	−0.476	0.003
	Total	116	−0.007	0.942	−0.359	<0.001	−0.287	0.002
Whole period	Mild	103	−0.365	<0.001	−0.288	0.003	−0.317	0.001
	Moderate	202	−0.512	<0.001	−0.64	<0.001	−0.652	<0.001
	Severe	86	−0.275	0.01	−0.748	<0.001	−0.742	<0.001
	Total	391	−0.338	<0.001	−0.476	<0.001	−0.462	<0.001

*We calculated the Spearman’s rank correlation coefficient between N antigen levels and SARS-CoV-2 IgM, IgG or NAb titers in the serum. rs, Spearman’s rank correlation coefficient*.

* One sample was not available to measure SARS-CoV-2 NAb titer because of short volume in the sample.

## Discussion

Until now, the clinical usefulness of serum N antigen testing in the diagnosis of COVID-19 and the associations of the serum N antigen levels with the clinical phenotypes of COVID-19 remain to be clearly established. To the best of our knowledge, only two reports have demonstrated the potential usefulness of measurement of the serum N antigen levels in the diagnosis of COVID-19 and the possible associations of the serum N antigen levels with the severity of COVID-19 (Deng et al., 2021; Perna et al., 2021). However, neither of these studies demonstrated the diagnostic ability or association with the disease severity of the serum N antigen levels at various time-points after the onset of COVID-19 symptoms. Since the serum N antigen levels would be expected to change dramatically on a daily basis in the acute phase of COVID-19, it would be desirable to take into account the time-point of measurement after symptom onset to determine the diagnostic ability/association with the disease severity of the serum N antigen levels.

Although asymptomatic cases were not included in this study, measurement of serum N antigen levels might be useful for the diagnosis of symptomatic COVID-19, since N antigen was detected in the sera of not only patients with severe COVID-19 but also in patients with mild disease, who did not require oxygen supplementation (Figure 1). According to a systematic review conducted by Böger et al. (2021), the sensitivity of RT-PCR is 73.3% (95% Cl 68.1–78.0%) for nasopharyngeal aspirate or throat swab specimens, 97.2% (95% Cl 90.3–99.7%) for sputum specimens, 62.3% (95% Cl 54.5–69.6%) for saliva specimens, and 7.3% (95% Cl 4.1–11.7%) for blood specimens (Böger et al., 2021). In regard to antigen testing, according to one study, the sensitivity and specificity are 76.1% (range, 44.4–100%) and 100% when nasopharyngeal swab samples are used for the measurement using a CLEIA automated analyzer (Hirotsu et al., 2021). Another study showed a sensitivity and specificity of 77.8 and 99.6%, respectively, for measurement in saliva samples, using a modified cutoff value (Asai et al., 2021). In this study, the diagnostic ability for COVID-19 of serum N antigen levels measured in serum samples was deemed to be equal to or even superior to that of RT-PCR or antigen testing in other types of samples, when we used the optimal cutoff value for serum samples determined in the present study (Figure 2). The only false-positive test result in a non-COVID-19 case in this study could be attributable to the presence of an autoimmune disease, considering that the patient had allergic granulomatous angiitis. In the validation of the cutoff value, the overall concordance rate between RT-PCR tests in nasopharyngeal swab and serum N antigen tests shows that the most optimal cutoff value in days 1–4 after the COVID-19 symptom onset might be 0.5 COI, the optimal cutoff value in days 5–10 was 0.255 or 1.0 COI, and after day 11, the overall concordance rates decreased (Supplementary Table S3). These results suggested that the cutoff value for the diagnosis of COVID-19 should be determined, according to the days after the symptom onset.

In regard to the ability of the serum N antigen levels to predict the maximum disease severity of COVID-19, as shown in Figures 3A–E, the subjects with moderate and severe COVID-19 tended to show higher serum N antigen levels than the subjects with mild disease. As shown in Supplementary Figure S4, the increase of serum N antigen levels was prior to the progression of disease. These results were consistent with previous reports suggesting a possible association between the maximum disease severity and viral RNA levels in the blood (Eberhardt et al., 2020; Fajnzylber et al., 2020). The difference in the serum N antigen levels between patients with mild and severe disease may become apparent at an earlier time than the difference in the anti-SARS-CoV-2-specific antibody titers, which begin to increase almost 1 week after the onset of symptoms (Jin et al., 2020; Long et al., 2020). It is noteworthy that no difference in the serum N antigen level was observed between the patients with moderate and severe disease. Although further studies are necessary, these results suggest the possibility that the viral load might determine the disease severity in patients with more than moderately severe disease, and that factors other than the viral load, such as complications and the immunological state of the subjects might contribute to the determination of the disease severity in cases with severe COVID-19. In any case, the serum N antigen level may serve as an early biomarker for predicting the maximum disease grade of severe COVID-19; for example, when the serum N antigen levels were over 93.505 at days 5–6, the clinicians need to consider the further progression of the disease severity and may need to transfer the subjects to the hospital which can offer intensive care with mechanical respiratory ventilation.

Measurement of the serum N antigen levels is also expected to allow a better understanding of the pathogenesis of COVID-19, in addition to its possible clinical usefulness of this laboratory test. For example, we observed a negative correlation between the serum N antigen levels and SARS-CoV-2 antibody titers (Table 2 and Figure 4). Considering that the titer of antibodies might be higher in subjects with a higher viral load, the present inverse correlation between the serum N antigen levels and antibody titers might possibly be a result of the antigen levels remaining at high levels in subjects with lower antibody titers, which might be insufficient for rapid clearance of the virus. Actually, this inverse correlation may be consistent with a previous study demonstrating that the production of antibody containing neutralizing antibody is delayed in patients with severe disease (Lucas et al., 2021).

This study had several limitations. First, since the study was a retrospective cross-sectional study, a prospective longitudinal study is necessary in the future to confirm the results of the study. Second, to validate the usefulness of serum N antigen measurement in the diagnosis and severity prediction of COVID-19, a study comparing the serum N antigen levels with the viral load determined by RT-PCR or the N antigen level in respiratory tract specimens collected at the same time as the blood specimens would be desirable. Finally, in the present study, we measured the serum N antigen levels only in symptomatic subjects who were hospitalized and could not elucidate the usefulness of serum antigen level measurement in asymptomatic cases or in the general population. Nonetheless, we believe that the present study might help us to understand the potential usefulness of serum N antigen measurement in the management of COVID-19.

In conclusion, we demonstrated the usefulness of measuring the serum levels of the SARS-CoV-2 N antigen using a fully automated analyzer. Serum N antigen measurement showed a high sensitivity and specificity for the diagnosis of COVID-19 and might serve as an early predictive marker of the maximum disease severity of COVID-19. Serum N antigen measurement may also contribute to a better understanding of the pathogenesis of COVID-19, such as by showing the negative correlation between serum N antigen levels and SARS-CoV-2 antibody titers, revealed for the first time in the present study.

## Data Availability Statement

The original contributions presented in the study are included in the article/Supplementary Material, further inquiries can be directed to the corresponding author.

## Ethics Statement

The studies involving human participants were reviewed and approved by the University of Tokyo Medical Research Center Ethics Committee. The patients/participants provided their written informed consent to participate in this study.

## Author Contributions

RY participated in the experiments and data analysis and drafted the initial manuscript. MK participated in the study design and helped in drafting the manuscript. YN and YM participated in the experiments. HO participated in the data analysis and visualization. CQ, FX, FH, LZ, YYu, YK, and JO developed the antibody measurement system. MM, YH, and SH participated in the data analysis and the discussion and helped in drafting the manuscript. DJ, KO, KM, and TK participated in the discussion and helped in drafting the manuscript. YYa conceived the study, coordinated the study design, and helped in drafting the manuscript. All authors contributed to the article and approved the submitted version.

## Funding

This work was supported by the Research Grants in the Natural Sciences from the Mitsubishi Foundation.

## Conflict of Interest

The present study was a collaborative research project among the University of Tokyo, Shenzhen YHLO Biotech Co., Ltd., and Medical & Biological Laboratories Co., Ltd. FX, FH, LZ, and YYu are the employees of Shenzhen YHLO Biotech Co., Ltd., and YK, JO, and HO are the employees of Medical & Biological Laboratories Co., Ltd.

The remaining authors declare that the research was conducted in the absence of any commercial or financial relationships that could be construed as a potential conflict of interest.

## Publisher’s Note

All claims expressed in this article are solely those of the authors and do not necessarily represent those of their affiliated organizations, or those of the publisher, the editors and the reviewers. Any product that may be evaluated in this article, or claim that may be made by its manufacturer, is not guaranteed or endorsed by the publisher.

## Footnotes

^1^ http://lab-tky.umin.jp/clinical_research/2019300NI-4.pdf

^2^ https://plaza.umin.ac.jp/utid/img/2020206NI8.pdf

## Abbreviations

ACE2: Angiotensin converting enzyme 2
AU/mL: Arbitrary units per milliliter
AUC: Area under the curve
CLIA: Chemiluminescence immunoassay
COI: Cutoff index
Cl: Confidence interval
COVID-19: Coronavirus disease 2019
Ct: Cycle threshold
N antigen: Nucleocapsid antigen
NAb: Neutralizing antibody
RBD: Receptor binding domain
RLU: Relative light units
RT-PCR: Reverse transcription polymerase chain reaction
ROC: Receiver operating characteristic
SARS-CoV-2: Severe acute respiratory syndrome coronavirus 2
