# Supplementary material for: Association of the Serum Levels of the Nucleocapsid Antigen of SARS-CoV-2 With the Diagnosis, Disease Severity, and Antibody Titers in Patients With COVID-19: A Retrospective Cross-Sectional Study
Source: Front Microbiol. 2021 Dec 9;12:791489. doi: 10.3389/fmicb.2021.791489 (PMC8696188; doi:10.3389/fmicb.2021.791489)
Supplement: Supplementary file 1 [file Image_1.PDF]

Supplemental Figure S1

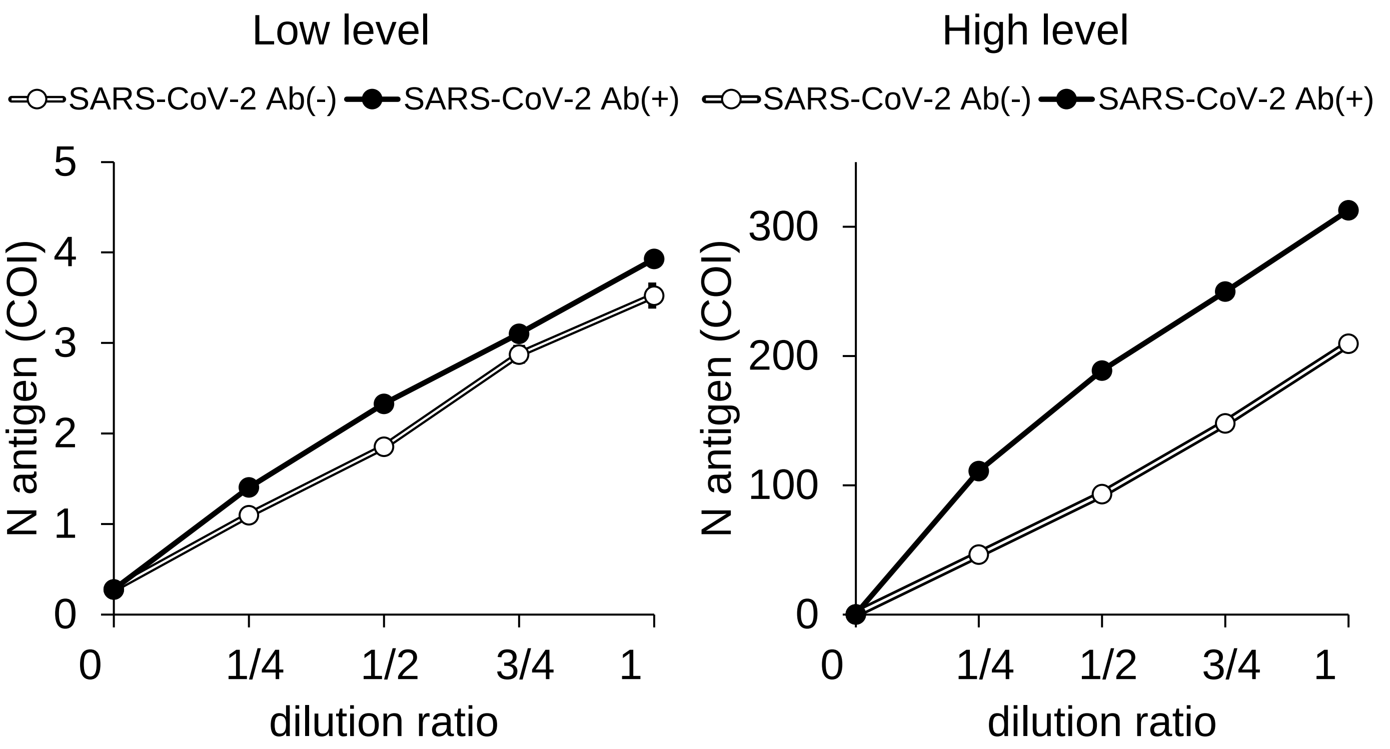

Supplemental Fig. S1. Linearity analyses of N antigen assay

The dilution linearities of N antigen assay was investigated. The pooled serum collected in 2017 was used as diluent. The serum samples collected from the COVID-19 confirmed patients, who were administrated immunosuppressive durg or anti-CD20 antibody drug to treat their chronic disease, were used as SARS-CoV-2 Ab(-). These antibody titers of the samples were described at Supplemental Table S2.

Supplemental Figure S2

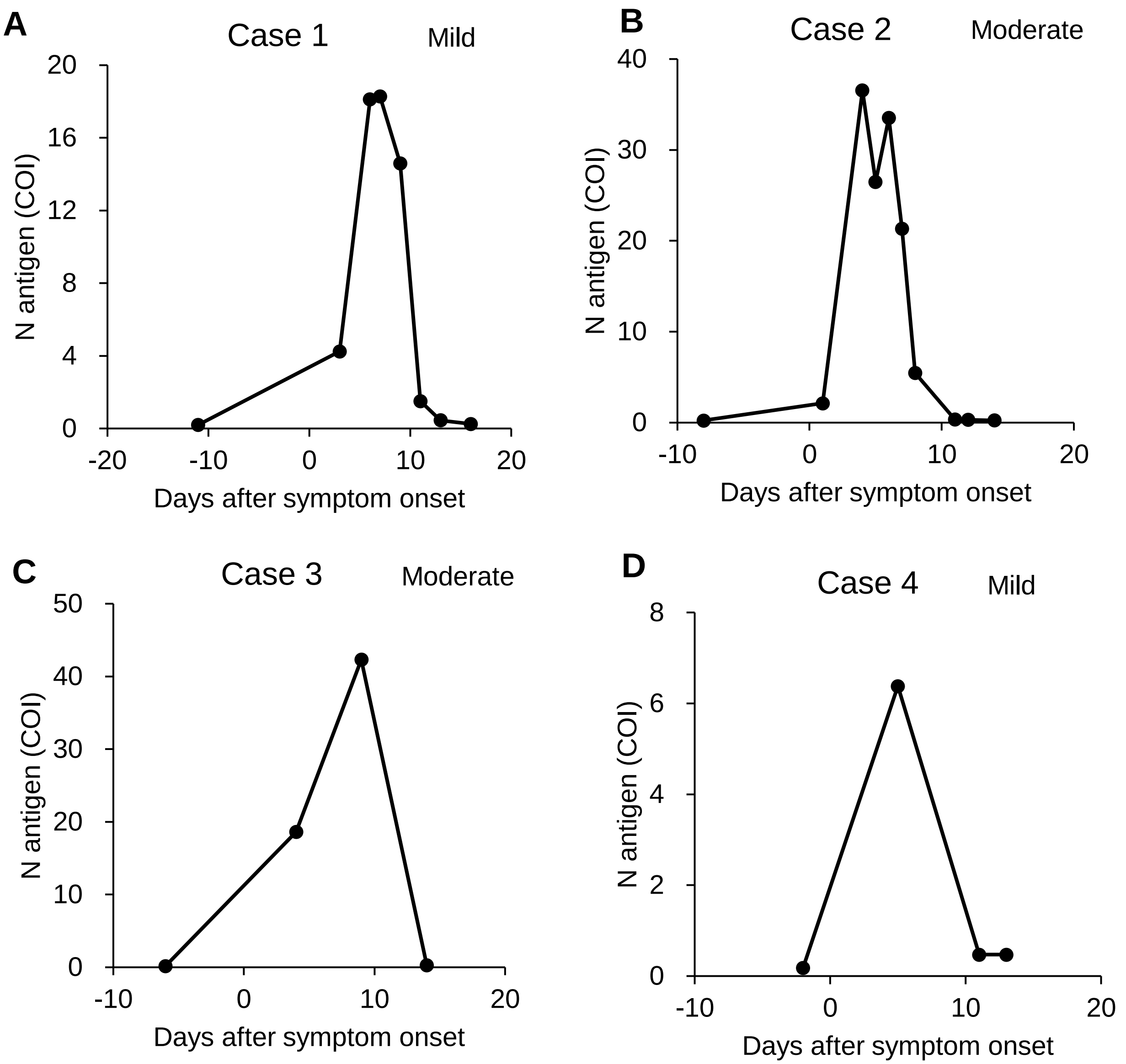

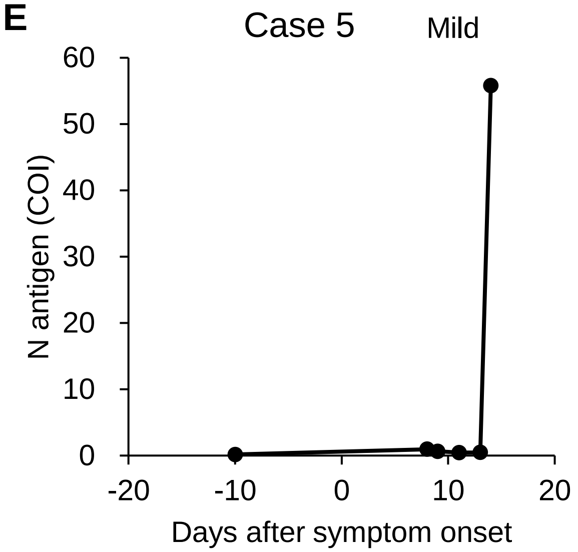

Supplemental Fig. S2. Time-courses of the serum N antigen levels in individual cases. Time-courses of the serum N antigen levels individually for the five cases illustrated in Fig. 1A.

Supplemental Figure S3

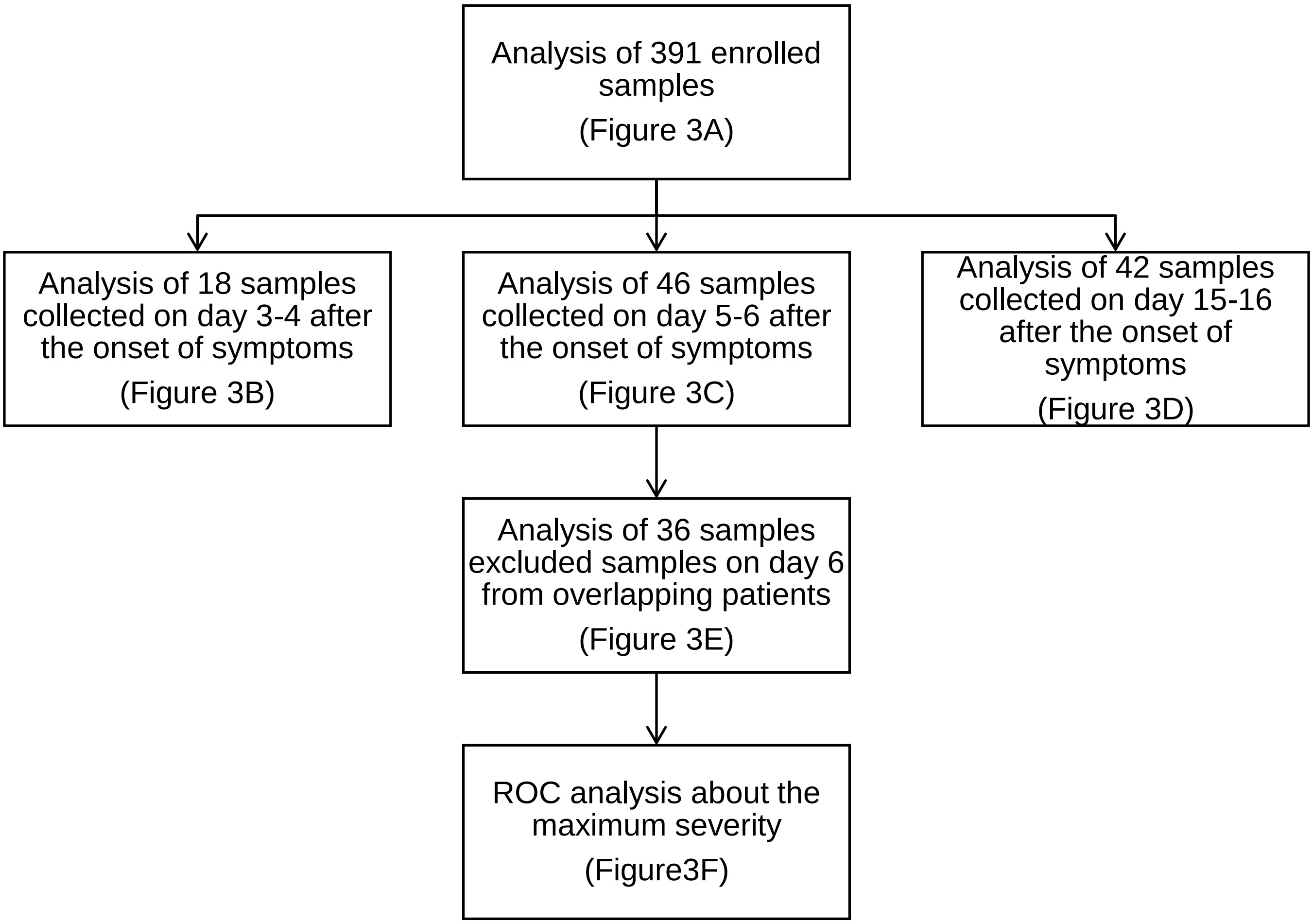

Supplemental Fig. S3. The flowchart of selection of samples used for the analyses on the association between serum antigen levels and the disease severity.

Supplemental Figure S4

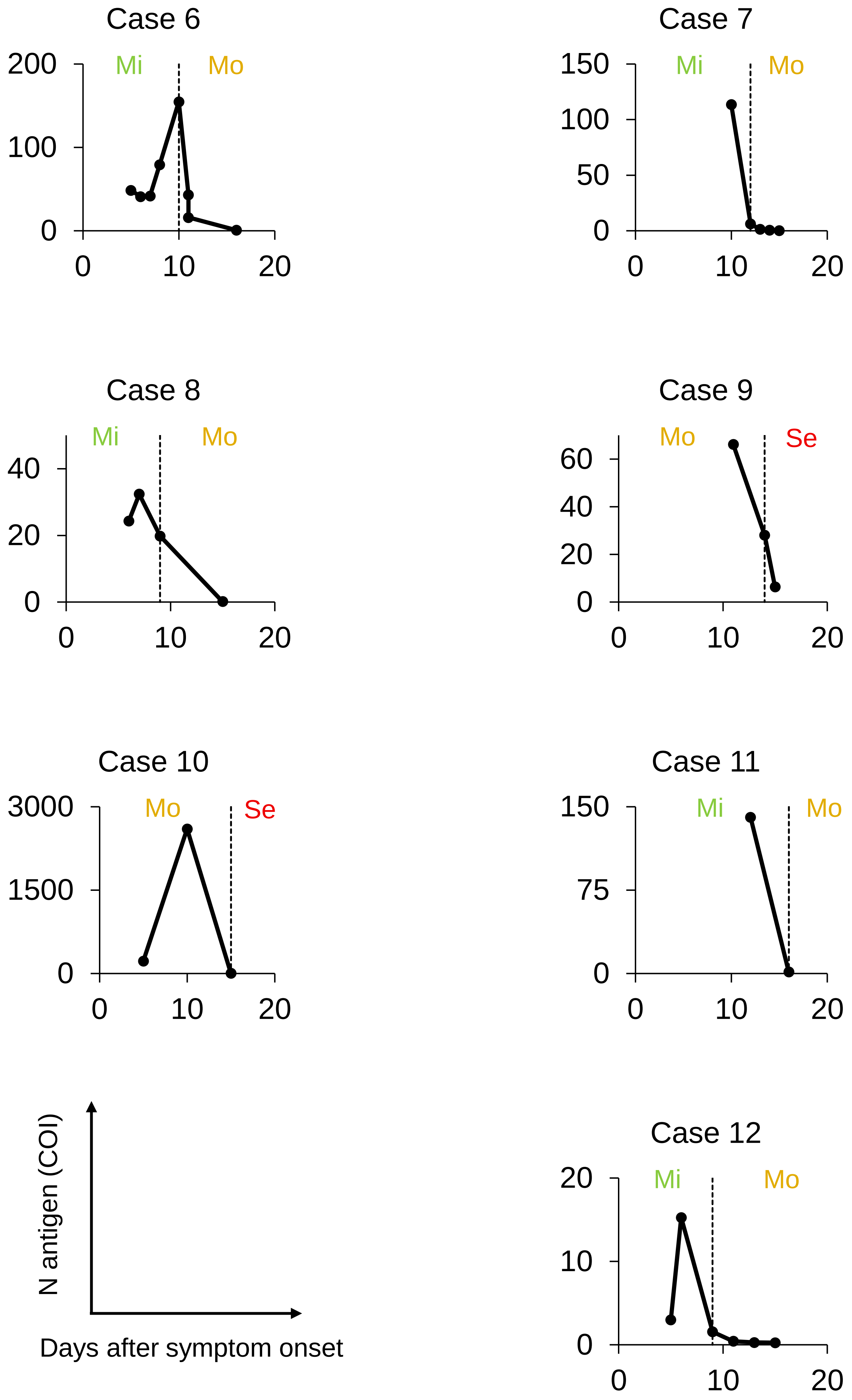

Supplemental Figure S4 (Continued)

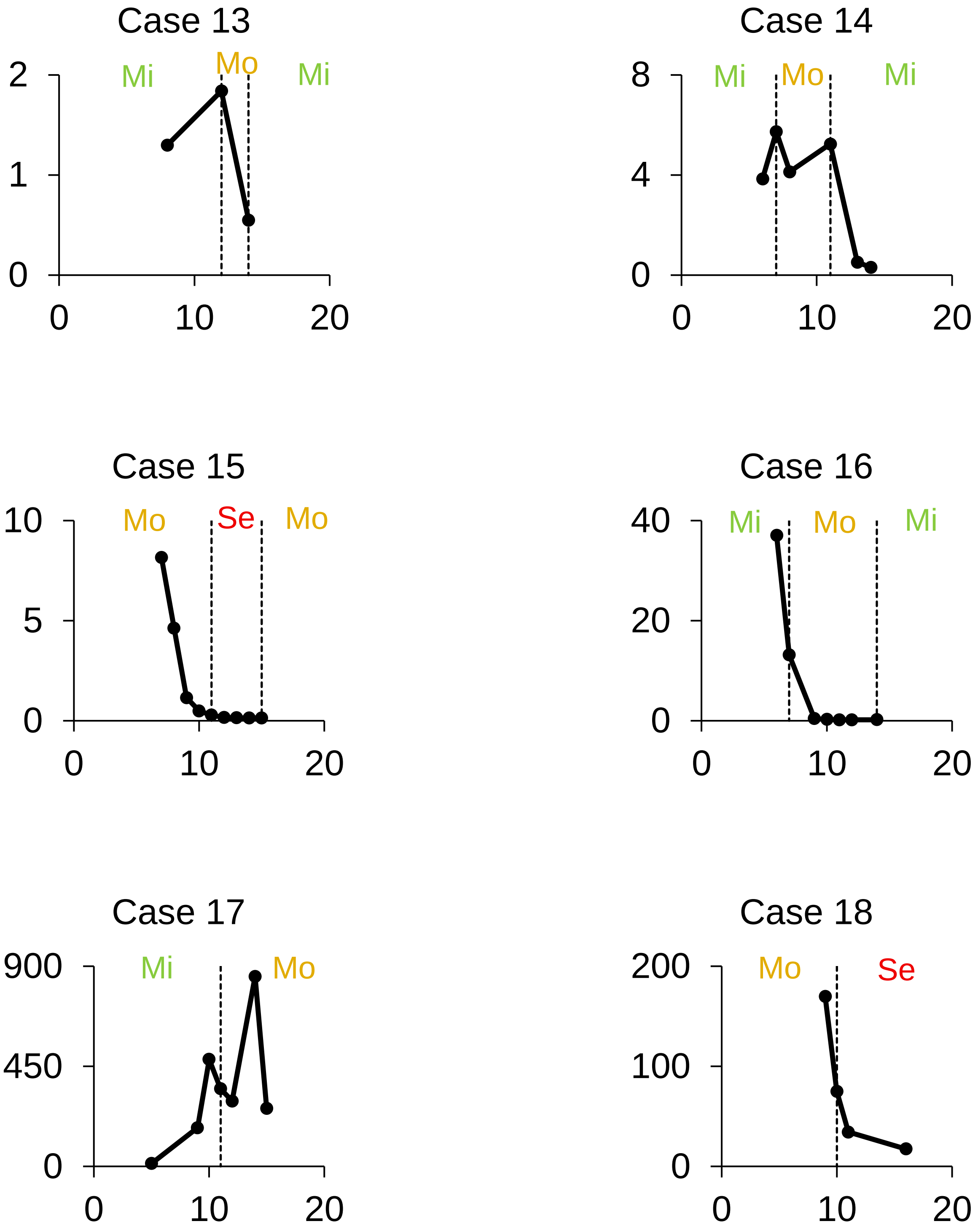

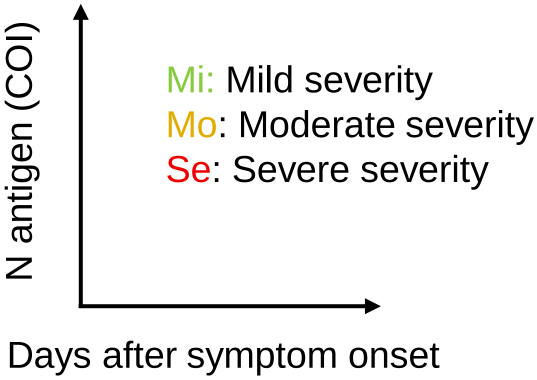

Supplemental Fig. S4. Time-courses of the serum N antigen levels and the progression of disease in individual cases

Time-courses of the serum N antigen levels and severity for the 13 individual cases in which the disease severity progressed between 1-16 days after the symptom onset.
