# Supplementary material for: Association of the Serum Levels of the Nucleocapsid Antigen of SARS-CoV-2 With the Diagnosis, Disease Severity, and Antibody Titers in Patients With COVID-19: A Retrospective Cross-Sectional Study
Source: Front Microbiol. 2021 Dec 9;12:791489. doi: 10.3389/fmicb.2021.791489 (PMC8696188; doi:10.3389/fmicb.2021.791489)
Supplement: Supplementary file 2 [file Table_1.PDF]

Supplemental Table S1. Characteristics of the subjects.

		COVID-19	non-COVID-19
Subjects [number]		101	96
Median of age [years] (%)		63.0 (51.0-72.0)	64.5 (53.0-74.5)
Female [number] (%)		34 (33.7)	32 (33.3)
Maximum severity [cases] (%)	Mild	33 (32.7)	
	Moderate	49 (48.5)	
	Severe	19 (18.8)	
Samples [number]	Day 1	3	
(Days after COVID-19 symptom onset)	Day 2	2	
	Day 3	7	
	Day 4	11	
	Day 5	25	
	Day 6	21	
	Day 7	27	
	Day 8	32	
	Day 9	34	
	Day 10	34	
	Day 11	44	
	Day 12	35	
	Day 13	36	
	Day 14	38	
	Day 15	27	
	Day 16	15	
	Total	391	96
Samples [number] (before the symptom onset)		5	

Supplemental Table S2. The samples used in the linearity test.

		N antigen (COI)	SARS-CoV-2 IgM (AU/mL)	SARS-CoV-2 IgG (AU/mL)
Diluent serum		0.28	0.39	1.35
SARS-CoV-2 Ab (-)	Low level	3.53	0.12	0.34
	High level	209.57	0.27	0.94
SARS-CoV-2 Ab (+)	Low level	3.93	12.02	93.42
	High level	312.69	2.97	36.19

Serum N antigen levels and SARS-CoV-2 antibody levels in the samples used in the linearity test described in Supplemental Figure S1.

Supplemental Table S3. The concordance rate with RT-PCR test

		Cutoff value			
Days after symptom onset	N	0.2 COI	0.255 COI	0.5 COI	1.0 COI
Day 1-4	28	86%	89%	93%	82%
Day 5-10	73	86%	89%	86%	89%
Day 11-16	22	73%	73%	73%	64%

We investigated the overall concordance rate between the serum N antigen measurement and the RT-PCR test for SARS-CoV-2 RNA, modifying the cutoff values of serum N antigen levels. The formula which was used to calculate overall concordance rate was described below. The RT-PCR samples which were collected on the same or ±1 day of collecting the serum samples were used for the analysis.

$$Oc = \frac{Pc + Nc}{N}$$

Oc = Overall concordance rate

Pc = Number of samples of positive concordance

Nc = Number of samples of negative concordance

N = Total number of samples used for analysis
